# The use of technology in the context of frailty screening and management interventions: a study of stakeholders’ perspectives

**DOI:** 10.1186/s12911-019-0828-6

**Published:** 2019-06-11

**Authors:** Holly Gwyther, Lex van Velsen, Rachel L. Shaw, Barbara D’Avanzo, Maria Bujnowska-Fedak, Donata Kurpas, Katarzyna Szwamel, Jan-Willem van’t Klooster, Carol Holland

**Affiliations:** 1 The Centre for Ageing Research, Lancaster University, Lancaster, LA1 4YG UK; 2 Roessingh Research and Development, Roessinghsbleekweg 33B, 7522 Enschede, AH Netherlands; 3 Research Centre for Healthy Ageing, School of Life and Health Sciences, Aston University, B4 7ET, Birmingham, UK; 4 Laboratory of Quality Assessment of Geriatric Therapies and Services, Istituto di Ricerche Farmacologiche Mario Negri IRCCS, Milan, Italy; 5 Family Medicine Department, Wroclaw Medical University, Syrokomli 1, 51-141 Wroclaw, Poland; 6 Opole Medical School, ul. Katowicka 68, 45-060 Opole, Poland; 7 BMS Lab, Faculty of behavioural, management and social sciences, University of Twente, Drienerlolaan 5, 7522 Enschede, NB Netherlands

**Keywords:** Older adults, Frailty, Disease management, Technology, Educational technology

## Abstract

**Background:**

Health and social care interventions show promise as a way of managing the progression of frailty in older adults. Information technology could improve the availability of interventions and services for older adults. The views of stakeholders on the acceptability of technological solutions for frailty screening and management have not been explored.

**Methods:**

Focus groups were used to gather data from healthy and frail/pre-frail older adults, health and social care providers, and caregivers in three European countries – Italy, Poland and UK. Data were analysed using framework analysis in terms of facilitators or determinants of older adults’ adoption of technology.

**Results:**

Our findings clustered around the perceived value; usability, affordability and accessibility; and emotional benefits of frailty screening and management technology to stakeholders. We also noted issues relating to social support, previous experience of technology and confidence of stakeholders.

**Conclusions:**

Professionals and caregivers understand the benefits of technology to facilitate frailty care pathways but these views are tempered by concerns around social isolation. Frail older adults raised legitimate concerns about the accessibility and usability of technology, specifically around the potential for their personal information to be compromised. Solutions must be developed within a framework that addresses social contexts and avoids stigma around frailty and ageing.

## Background

Frailty is a state of health often related to the ageing process, during which people gradually lose their psychological and physical reserves [1]. The development of frailty often goes unnoticed and the loss of reserves results in older adults becoming less resilient to stressors, which may escalate, leading to hospitalisation, loss of independence and death [2–4]. Although there is no benchmark definition of frailty, many operational definitions have emerged over the past two decades, with two highly cited assessment tools being Fried’s phenotype [5, 6] and the accumulation of deficits model [7, 8]. Recent evidence suggests that frailty is a dynamic and transitional process and that there may be opportunities to reverse, manage or prevent its progression through intervention [9, 10].

The early identification of pre-frail and frail older adults through population screening programmes may provide an opportunity to effectively target interventions to better manage frailty and improve health and wellbeing [11, 12]. Shaw et al., [13] established and Gwyther et al., [14] supported the view that frailty screening programmes would be considered a positive contribution to older adults’ health care by stakeholders including frail older adults and health care policymakers, as long as they directed them to an outcome or treatment, and did not simply classify or label them as frail.

The incorporation of information technology solutions into frailty screening and management interventions could help health and social care providers to deliver clinically valuable and cost effective solutions to improve older adults’ quality of life and wellbeing. It has been suggested that the use of various information and communication technologies (ICT) such as mobile telephones, home computers and the Internet could improve quality of life and reduce health care costs for older adults generally [15] and improve communication and information transfer between professionals and patients [16–18]. There are further suggestions that technology could positively affect frailty status [19]. As a result, van Velsen [20] developed a comprehensive online service (PERSSILAA: Personalised ICT Supported Service for Independent Living and Active Ageing) to screen older adults for pre-frailty and to attempt to improve the health of those who were classified as pre-frail (defined as functional decline) via online services, focusing on physical and cognitive training, and nutritional information. Evaluation of the physical training showed that it was easy to use and has the potential to improve quality of life and the older adult’s functional health status [21]; online screening of the frailty status of older adults, measured using scales for sarcopenia, physical functioning and quality of life, resulted in a reliable clinical assessment [22].

Although technology offers an opportunity to manage health care costs and enhance the lives of older adults [23], historically the rates of computer use are low in this age group compared with other age groups. Previous studies have found that older adults who use computers are likely to be ‘younger’ (e.g., [24, 25]), male (e.g., [25, 26]), with higher levels of education [25] and income [27] and with fewer health problems or functional impairments (e.g., [17, 25, 28]). Recent figures from a study investigating the level of Internet diffusion in the older European population using data from the Survey of Health, Ageing and Retirement in Europe (SHARE: [26]) noted that an average of 49% of all respondents used the Internet. However, the historical demographic divides by age, gender and socio-economic background still held true. Further, there were geographical differences across Europe in Internet usage, with a reported Northwest European slope. For example in Poland and Italy 33 and 35% respectively of older adults reported using the Internet, while in Denmark the figure was around 83%. UK data was not examined in this study but recent analyses [29] show that although Internet usage has trebled amongst women and those aged over 75 years since 2011 only 41% of this age group are regularly online.

The reasons for the ‘digital divide’ [30] and the barriers to Internet and technology usage amongst older adults in healthcare have been explored in the literature (e.g. [17, 18, 27, 31]). Amongst this literature, Lee and Coughlin [32] describe a general and holistic framework which identifies ten facilitators or determinants of older adults’ adoption of technology: value, usability, affordability, accessibility, technical support, social support, emotion, independence, experience, and confidence. These factors are further described in Table 1. These authors suggest that in order to design and develop a technological tool for older adult consumers, the whole person context should be considered, including their individual characteristics and social environment. This assertion is shared by Peek and colleagues [33], who further stated that the acquisition of technology by older adults is a circular process, where past experiences affect subsequent technology adoption decisions. Lee and Coughlin’s [32] theoretical paper on technology use for older adults was used to frame our analyses and was chosen because of its comprehensive approach to examining the barriers in technology use but more importantly for the development of future health technology solutions, it also incorporates practical applications and facilitators of use.

**Table 1:** Factors of Older Adults’ Technology Adoption ([32], p750)

Factor	Description
Value	Perception of usefulness and potential benefit
Usability	Perception of user friendliness and ease of learning
Affordability	Perception of potential cost savings
Accessibility	Knowledge of existence and availability in the market
Technical support	Availability and quality of professional assistance throughout use
Social support	Support from family, peers and community
Emotion	Perception of emotional and psychological benefits
Independence to others	Perception of social visibility or how a technology makes them look
Experience	Relevance with their prior experiences and interactions
Confidence	Empowerment without anxiety or intimidation

Certainly, for technologies associated with frailty management to be efficient and effective, stakeholders in frailty must be willing and able to use them. Stakeholders are all the people or organisations that have a task or role in relation to, or are affected by the technological intervention [34]. In the context of frailty, besides older adults (who are, in most cases, direct end-users), other stakeholders also play a crucial role in implementing technology. They could be as diverse as General Practitioners (GPs), occupational therapists and physiotherapists, nursing staff, informal caregivers, municipalities and healthcare insurers. Even though these stakeholders might not benefit the most from the technology, its goals and functionalities should fulfil a need for them (or should, at least, not act against their values and wishes), as they can be paramount for financing or handling of escalations of care when required. Strong involvement while developing, evaluating and implementing eHealth solutions is of great importance for its success [35, 36]. However, while the involvement of prospective end-users is becoming common practice when developing and implementing eHealth technology, involving stakeholders is still quite rare.

Understanding and clarifying the issues faced by older adults and the set of wider stakeholders in the care of older adults is essential in determining the acceptability of health-related ICT for frailty screening and management purposes in older adults, and the way in which it might be implemented. Therefore, the aim of this study was to determine stakeholders’ views about the adoption of health-related technology for frail older adults, specifically for the purposes of frailty screening and management, including the delivery of interventions designed to reduce or manage the progression of frailty.

## Method

This study forms part of a wider range of studies known collectively as FOCUS [37, 38]. This study reports a secondary analysis of data collected from focus groups with stakeholders in three countries - Italy (Milan), Poland (Wroclaw) and the United Kingdom (Birmingham). The primary findings from across all three countries relating to frailty screening and management [13] have previously been published. Additional findings relating to accessibility to health care in Poland [39] have also been published. This paper focuses solely on findings related to the acceptability and adoption of health-related technology solutions for frail older adults, specifically for the purposes of frailty screening and management, including intervention delivery.

### Participants and recruitment

We conducted semi-structured focus groups and qualitative interviews with key stakeholders, including frail and robust older adults, health care professionals, family caregivers and social caregivers. Stakeholders were recruited through purposive sampling. The sample characteristics and recruitment strategies have been previously described [13, 14, 39]. Briefly, older adult participants were sourced through invitations to a research centre volunteer panel, through advertisements in social centres including recreational centres, churches, schools, older adult education and learning facilities, retirement villages, and in General Practitioner clinics. Individuals were included as long as they had mental capacity to consent. Participants were defined as being frail (*n* = 28) or robust (*n* = 23). In the UK frail participants were identified using a measure based on an accumulation of deficits model, including physical, cognitive and social measures [7]. In Italy and Poland, participants self-identified as frail or not, using the information sheet given to each participant, a view which was confirmed by a physician’s clinical judgement. Health (*n* = 26) and social care professionals (*n* = 22) were required to be in an active role with at least two-years’ service. They were recruited through professional networking and social care centres. Those recruited included general practitioners, nurses, clinical psychologists, occupational therapists and physiotherapists, as well as care workers and social workers. Caregivers (*n* = 19) were recruited through health and social care services, as well as through patients’ associations. They were required to provide care and support for a frail older adult on a regular basis but not necessarily co-reside.

### Data collection

Eight focus groups were conducted. Less mobile participants who wished to participate were offered the option of a home interview and three were conducted. Discussions with older adults and caregivers were held in non-medical settings in Poland and the UK but in a hospital in Italy. All data collection occurred between October 2015 and January 2016 in participants’ native languages. Focus groups (*n* = < 11 participants) and interviews were conducted separately for each stakeholder group and lasted between 11 (where the participant became distressed) and 65 min in the UK; between 60 and 130 min in Italy; and between 48 and 90 min in Poland. Focus groups were facilitated by female researchers in all countries, in Italy by a senior researcher and a psychologist with experience of qualitative studies, in Poland by two General Practitioners, and in the UK by a psychologist. Researchers were not known to the participants and no personal information was provided.

Two semi-structured questions were used which were defined in advance and identical for all three countries. These questions concentrated on the views and experiences of older adults in relation to technology, but specifically about using the Internet, computer or online tools for frailty screening, as well as for physical and cognitive exercise purposes. The questions were:

1. ‘Imagine you could assess your frailty status via a set of questionnaires on a website. How would you feel about this?’
2. ‘Imagine that you could train your health in order to reverse frailty or to prevent it via a website, for example by watching exercise videos on a website that show you how you can train your body to increase your strength, or doing exercises to improve your mind. Would this be something you’d be interested in?’

Questions were directly posed to all stakeholder groups during the discussions. All discussions were digitally audio-recorded and transcribed verbatim in their language of origin. For practical reasons, only pertinent quotations were translated into English to enable comparison across countries.

### Data analysis

Pertinent quotations were extracted from the transcripts by local researchers, translated into English and collated by the primary analyst, a psychologist experienced in qualitative research and applied health research with older adults (HG). The data were then synthesised using framework analysis [40]. Framework analysis is a five-stage process involving: familiarisation with the data; identifying a thematic framework; indexing responses; reviewing and revising the framework; and mapping and interpretation of themes. Given the large body of literature on older adults’ adoption of technology, we considered that a deductive approach to the data would be appropriate. Lee and Coughlin’s [32] theoretical paper on technology use for older adults was used to organise the data. Data were categorized according to the ten facilitators or determinants of older adults’ adoption of technology: value, usability, affordability, accessibility, technical support, social support, emotion, independence, experience, and confidence. The framework was discussed with the other analysts, quotations studied and understandings confirmed with native language speakers. The Critical Appraisal Skills Programme (CASP: 2017) Qualitative Research Checklist was used to guide reporting.

### Ethics

Ethical approvals from each participating locality were granted. Volunteers were given participant information sheets in plain language and written informed consent was received.

## Results

Ten factors were reviewed but only nine are described: *value, usability, affordability, accessibility, social support, emotion, independence, experience,* and *confidence* (Lee and Coughlin, 2014). The tenth theme, *technical support* was not supported by evidence in this study. Technical support relates to the availability and quality of professional assistance throughout use of the product. Given that specific ICT tools for frailty are not in widespread use, it was unsurprising that participants did not describe this factor. Each theme is described briefly and example quotations are shown in Table 2. Quotations are attributed by country, participant group and participant number.

**Table 2:** Representative quotations for each factor (after [32])

Factor	Example quotations
Value	*Suppose there was a paper copy of what was on the website as well, because there is this assumption that everybody wants to go to a computer. Sorry, there are still groups of us who don’t necessarily automatically do that…..So I think your consensus round here is that it might be a very good idea [screening] but don’t just think technical.* [UK Robust Older Adult 13] *The way I was thinking about it just then was to see it as being most useful in primary care but would be something that would be part of their records which would be accessible if they were to go in to hospital, you’d have more of an idea. If that information were available, not just a score but […] a quick snapshot of what the vulnerabilities were, that would be incredibly useful information […]. I can’t see, the idea of doing it online, yeah that would be great for those who aren’t actually frail but I think for those who are, then you are actually going to be missing out on a lot of the people with greatest need because they’ll have visual problems or they’ll have anxieties about computers and stuff like that. I don’t see that as being terribly helpful*. [UK Health care professional 30] *I think you’d be able to target services if you were all singing from the same hymn sheet because we have difficulties at times identifying urgency and priority and being able to respond but if you were told, this person’s got a frailty score of 4 and that was quite high, right okay, we’ll get out, we’ll do that intervention* [UK Health care Professional 21] *We need computer systems that talk to people, that highlight … and if we were able to get more savvy…., it would reduce duplication for some services, get in at the right time, get that quick intervention to get them back on the even keel* [UK Health care Professional 21] *Perhaps training for some caregivers, but also a website, because not only do they work and can’t make it sometimes, but they also have little time for their family as they are preoccupied by that elderly person. How are they supposed to go for training? It’s a problem. Nothing against training for the elderly.* [Poland Healthcare Professional 7]
Affordability, Accessibility and Usability	*We’d be lucky if they have a computer.* [UK Health care Professional 20] *From an Internet point of view, most of our patients don’t have access to the Internet at home.* [UK Health care Professional 18] *I do not have a computer.* [Italy Robust older adult 1] *Yes, I can use a computer but I don’t own one, I go in the library. I mean no library now is open at nine o’clock. If it was, I’d probably pop in and see if I could do it but there again, they’re on a timer and after half an hour they go off, they’re on a timer. You might get two hours, I’d have to mention it. No, the libraries don’t open until nine, ten at night* [UK Frail Older Adult 2] *Everybody’s got a television. All the old people have got televisions. If you had a keep-fit on television, I think that might be a good, […] but it’s a good thing to have, exercise, it’s a good hour of exercising, in a chair mostly and because it’s a physio doing it, it’s not doing you any harm.* [UK Frail Older Adult 3] *Internet no.. we need to wait a few years. Nobody uses it* [Italy Family Caregiver 5] *I have got a mobile, I take it with me, but I cannot use it.* [Italy Robust older adult 11] *We didn’t have a mobile phone, somebody had made an assumption that everybody has a mobile phone* [UK Frail Older Adult 12]
Social support	*No. so that couldn’t be something that would be easily accessible and often a lot of our patients do have a cognitive problem so they wouldn’t remember to practice the exercises on their own. They would need to rely on carers or family to practice them with them, in order to notice any difference.* [UK Health care professional 18] *I don’t know because I put the exercise DVD on and I never do it.* [UK Social care professional 6] *You know my Father had Parkinson’s and he had exercises that were shown to him by a physiotherapist and whilst he was seeing the physiotherapist he maintained them but it was quite difficult because after they stopped, Mum did her best, she tried to do the ‘elephant arms’ and things every day but it’s much harder to maintain that when you need to do it on your own.* [UK Health care professional 30] *After retiring I went to computer classes. My husband did not feel like it, but I felt like going. I have got six grandchildren, and they help me.* [Italy Robust older adult 4]
Emotion	*These days, the Internet’s good for lots of things but it is bad for this, because it isolates people.* [UK Family Caregiver P15] *There are many people who use the computer and just get isolated.* [Italy Robust older adult 11] *P12: ….You know, you’re thinking about the computer, there’s a lovely example in this place, we were in car park 12 and as usual the parking meters, the pre-payment meters weren’t working and there was a new notice up telling you to get on your mobile phone and pay that way. Now a) we didn’t have a mobile phone, somebody had made an assumption that everybody has a mobile phone and b) even if we had got a mobile phone we wouldn’t make a financial transaction using a mobile phone. You know there’s a lot of assumptions made in different places about different things by….*[UK frail older adult 12] *I hate it, even when it is simplified* [Italy Robust older adult 9]
Experience and Confidence	*In addition, I think that such a website is a nice thing. For example, I have some rather old patients suffering from insomnia, and they say: “Doctor, if not for the Internet, I’d have long gone crazy. When I wake up, I can always browse or read something.”* [Poland Healthcare Professional 7] *I mean I find forums quite useful things* [UK Robust older adult 1] *Television makes me sleep and the computer I cannot use it* [Italy Frail older adult 9] *No, we’re too old for that [using a computer]!* [UK Frail older adult 26] *I struggle to use one [a computer]* [UK Frail older adult P10] *It depends on their state of mind doesn’t it? Some people, yes, they’d probably be quite happy to do it, others would probably be like, well I’m eighty odd, I’ve lived my life, whatever’s going to happen, is going to happen. I’m just going to do what I want.* [UK Health care Professional 7] *Books and the Internet are my escape. I am glad that I still have good memory. My wife says that I have a computer in my head. You know, my mind is still functioning properly* [Poland Robust older adult 7]. *I can manage. I do not feel cut out. For instance, today the nurse has sent my test results with some indications.* [Italy Robust older adult 4]
Independence	*Well with carer support, I suppose like you said, if someone was sitting alone at home and they already felt like they didn’t want to go outside and they felt frail and vulnerable, giving them questions like “How many times do you go out a week?” and “Can you walk to the shops?” and they’re like “No, no” it might make them feel worse.* [UK Health care professional 29] *That’s what I think we are worried about because then that could feed in to yeah, further concrete thinking around “Wow, if that’s what the score says, I must be” and then kind of living according to that. It might feel quite trapping.* [UK Health care professional 28]

### Value

Value examines the perceived usefulness and potential benefit of technology to the end user. Stakeholders including older adults perceived that frailty screening could be a positive asset as long as that screening lead to a supported treatment or management plan. However, it was apparent from older adults that despite an acknowledgement of the benefits of screening programmes and a lack of objections to technological solutions in principle, the preferred default was not a technology-based screening tool, and indeed one participant suggested that ‘low-tech’ alternatives should be considered.

Social care professionals were less concerned with the specifics of *how* screening occurred (e.g. via online questionnaire or paper version) and more concerned with the *why,* i.e., the purpose of the screening and whether it would lead to an improvement in quality of life for their clients. However, health care professionals (including physiotherapists and clinical psychologists) recognised that there was value in the use of technology in frailty, specifically in terms of an online screening programme designed to quantitatively categorise frailty, which could then be used as a tool to prioritise and target services, and as a direct and unambiguous method of communication between stakeholders in the frail older adult’s life. Similarly, social care professionals in Italy, suggested that online frailty screening and interactive tools would be useful in disseminating information relating to frailty to the general public but that mediation would be necessary to ensure that stakeholders were provided with appropriate advice and ongoing referrals where necessary.

### Affordability, accessibility and usability

Affordability relates to the perceived costs of technology while accessibility in this context relates to its availability. Usability relates to perceptions around user friendliness and ease of learning. In this study, it was difficult to separate out the three factors. Ownership of, and access to technology was often related to perceived cost, lack of interest and reluctance towards learning how to use the technology. Therefore the three factors are considered together. Many of the frail older adults interviewed stated that they did not own a computer (generally perceived as a desktop or laptop but also including tablet) or a mobile phone, a finding that was confirmed by the health care professionals. However, many participants noted that they were able to access a computer, either through a family member or friend, or through community resources such as a public library. Although these were not always available at a convenient time.

The potential of physical exercise interventions to reduce or manage frailty was generally well received. In the UK, people saw greater value in these being developed and delivered by trained professionals, either in classes or through the medium of television, perhaps using DVDs, rather than an online format. Participants mentioned the ubiquity of televisions and their accessibility, over online formats.

In Poland, health care professionals suggested that an online training platform for frailty management could be developed. The idea was that this would be a convenient and accessible way of learning about frailty and any skills or new methods to manage frailty could be directed to individuals. Further, it would not require the frail older adult or their care partner to leave their own home. The view was that online training could prepare older adults for self-care while saving caregivers’ time.

### Social support

Social support relates to support from family, peers and the wider community. This was a key factor for stakeholders in this study. Health care professionals suggested that some older adults would need support in order to access online materials. This support might entail loaning mobile technology for access to online services or simply reminding people to participate in interventions where they had the technology available. When asked about the likelihood of older adults taking part in interventions online, one participant was dismissive of its potential suggesting that adherence and compliance to intervention regimes would be challenging. This issue was widely raised, with stakeholders noting the value of online exercise interventions but suggesting that participants might lack the motivation or skills to participate in them without social support.

However, the key thread running through findings was that online interventions might put older adults at risk, of both social isolation and injury. There were strong views that older adults would need something else, over and above the online guidance, they might require somebody to motivate and encourage them, or protect them from over-exertion, and that exercising in a social and supervised setting would be preferable.

### Emotion

Emotion relates to the perception of emotional and psychological benefits of the technology. This was a significant factor in stakeholders’ views of technology. For example, social care professionals saw no emotional or psychological benefits in screening technology per se*.* In fact, there was a strong belief that simply receiving a ‘frailty’ score online and being categorised as frail would be psychologically damaging.

Similarly, family caregivers were also concerned about the potentially negative emotional effects of online interventions. They were worried that an online format would result in a greater risk of social isolation and loss of psychological resilience.

There was also a certain amount of caution, perhaps even fear of using technology in certain circumstances, for some older adults. This was specifically expressed in terms of undertaking financial transactions on mobile devices but extended to sharing personal information.

Although health care professionals saw benefits in using technology to assist with maintaining health records, they were concerned about older adults having access to screening questions or health information as they felt that the questions or results might make patients vulnerable to depression or negative thoughts. Further, they were concerned about the abilities of older adults to undertake their own screening. However, they agreed that an online screening instrument could be a useful tool for carers to use, perhaps to prompt a doctor’s visit or to encourage discussion during a health check.

### Experience and confidence

Experience refers to older adults’ prior experiences and interactions with technology. Confidence refers to a person’s ability to use a technological tool without anxiety. These themes were again considered together. Few of the older adults had significant previous experience with technology and it is likely that this contributed to the general reluctance to use the Internet and computers. Generally, the stakeholders we spoke to, including older adults and their caregivers suggested that the Internet and computers were difficult to use. Further, they also suggested that they considered themselves ‘too old’ [UK Frail Older Adult P26] and were not interested in using technology. Some healthy older adults in Italy were marginally less negative about online screening tools but similarly suggested that they had difficulties using the Internet. They suggested that training could be provided through older adult organisations or associations, which would also provide an access point for other activities.

However, there were a few exceptions of people who enjoyed using technology and had either spent their careers working with technology or discovered it as a hobby in later life. These people were confident and open to the idea of technological tools for health care, with one UK-based healthy older adult suggesting that a robot would be preferable to a human being for his personal care needs in older age.

### Independence

The independence factor relates to social visibility and how technology makes a person look to others, for example whether it makes them appear dependent, frail or in need of special care. However, it is primarily concerned with preventing stigmatisation and protecting autonomy. This is challenging in this context. Certainly, there were views from health care professionals in the UK that being categorised as ‘frail’ would be detrimental to a person’s psychological health. This suggests that there might be a stigma or shame attached to using online tools or resources dedicated to frailty management, as first an older adult would need to admit that they were indeed either frail, or vulnerable to frailty. This stigmatisation might drive them away from adopting or using the technology, even in the event that it promoted autonomy and independence.

## Discussion

This paper examines stakeholders’ perspectives on the use of technologies for frailty screening and management technologies. Given the extensive literature on older adults’ adoption of technology, we used a deductive strategy to examine our data. Lee and Coughlin’s [32] theoretical paper on technology use for older adults was used to frame our analyses. It was chosen because of its comprehensive approach to examining the barriers in technology use but more importantly for the development of future health technology solutions, it also incorporates practical applications and facilitators of use. We were keen to use a balanced approach rather than focus solely on barriers or negative aspects. Although our findings fit comfortably within the framework, we extend the knowledge on older adults’ potential adoption of technology by examining the viewpoints of a range of stakeholders, including those who would support frail older adults in their use of proposed technologies and also by applying the framework to a specific issue, that of frailty screening and management.

Technological tools for frailty screening and management are still, with some exceptions, believed by stakeholders including frail and healthy older adults to be inaccessible, challenging to use and unnecessary. Although health and social care professionals note the benefits of online tools for frailty screening to facilitate care pathways and online interventions as a way of improving health status, these views are tempered by concerns about older adults’ ability to access the technology and the potentially damaging psychological effects of self-screening for frailty and increased social isolation.

To change stakeholders’ perceptions on the value of technology solutions for frailty management, they must first understand that frailty is not necessarily a fixed state [9, 10] and that there are things that people can do to improve, or at least to manage their health and quality of life. Being clear about the benefits of knowing your frailty status and how you can manage, and even ameliorate that may promote interest in technological solutions.

However, additional efforts beyond changing the outlook of older adults, are also required to foster the use of technology for frailty screening and management. There are issues of access. As in our study, Selwyn [27] noted that many older adults have access to a computer, through family, friends or community resources, even if they do not personally own one. However, this in itself creates barriers to access for intervention participation. Computers in community spaces may only be available during working hours, or may have time limits imposed for use. Similarly, relying on family or friends for goodwill to take part in activities could be challenging. There may also be constraints on the activities that people would be willing to participate in within a public space, for example, they would be highly unlikely to choose to exercise in the public library. Further, while we as researchers were considering the design of new applications for tablets and mobile phone technology, stakeholders within the study seemed to be thinking primarily of desktop or laptop computers. Clearly, there was a mismatch between our and some of our participants’ viewpoints here. The use of these more mobile technologies such as tablets or telephones could assist in overcoming some of the barriers raised by participants in this study. However, the issue of access remained as despite the view that these technologies are potentially ubiquitous, many of our participants did not own or use mobile telephones. It may be that more affordable and user-friendly devices, coupled with readily available training would help to promote and demystify technology use for frail older adults.

Even when people are prepared to engage with technology, there was a strong belief from stakeholders that a substantial level of social support would be required to achieve the full benefit of interventions. Health and social care professionals suggested that with individual variation in motivation and physical health, older adults would need to be supported through the process. On one hand, health care professionals were concerned that online interventions would result in poor compliance and low rates of adherence. This perception is supported by the literature as exercise interventions have been found to be effective at reducing frailty in older adults but only when conducted in groups [9], possibly through the increased commitment generated through social interaction [10]. However, on the other hand they also raised the new concern that older adults would over-commit to the intervention, potentially exerting themselves with exercise beyond safe limits given their existing capacity.

Family caregivers saw the potential for online home-based interventions, specifically the provision of health care information and advice, as a way of assisting them to manage their time and ensuring that they could engage with content in a way that fitted within their already busy and challenging lives. However, many of them were suspicious of online interventions for older adults and viewed them as more socially isolating than alternatives. Maintaining social interactions with others and considering the social context within an intervention for frailty are of critical importance [13]. Thus, our conceptualisation of frailty is such that social health makes up one component of the frailty triad with physical health and psychological health making up the other two components. Evidence suggests that there is significant interplay between the three components and that social engagement is necessary to build psychological resilience and reduce the likelihood of functional disabilities and adverse outcomes [41] or physical frailty [42]. Thus, health technology interventions for frailty should emphatically address the need for social interaction in older adults.

There was a strong objection to personalised online frailty screening. We envisaged that online frailty screening would be based around a sensitive, validated and reliable measure of frailty, utilising an accumulation of deficits type index incorporating social, physical and psychological components. However, health and social care professionals believed that self-screening online could lead to psychological damage and health decline. This perception taps into the notion that damaging psychological resilience subsequently diminishes other components of the frailty triad. This has been previously examined [41, 42]. Certainly, there is evidence that depression is a predictor of an increase in frailty [43]. We also note that specific aspects of psychosocial factors have been associated with changes in functioning in adults with other chronic diseases [44]. For example, Seeman and Chen et al., [44] found that greater emotional support independently predicted lower rates of functional decline in people with cardiovascular disease, and that self-efficacy beliefs were protective of declines in functioning in cancer survivors. Thus, the provision of psychological support or work on self-efficacy when receiving a ‘frailty diagnosis’ could be critical in maintaining psychological and subsequently physical resilience in frail older adults. Beukema et al., [45] investigated how screening results should be presented to older adults when they participate in online frailty screening. They found that when the outcome is positive, a simple message suffices. When an older adult is confronted with an outcome of him/her being frail or pre-frail, the message should be tailored towards personal characteristics and the personal medical situation. This increases acceptance of the message.

## Limitations

The evidence from this paper originates from a purposive study of stakeholders in three European countries, all with low to moderate level of Internet diffusion in older adults [26, 29] and findings may be different in countries where Internet diffusion and information technology literacy is higher. Further, the frail stakeholders in the three different countries were classified using different methods (using an accumulation of deficits model in the UK and self/physician classification in Italy and Poland). Although there is no ‘gold standard’ for frailty measurement, we accept that this difference may have provided different results in terms of frailty status. However, these findings remain valuable and are transferable across technology-based interventions designed to reduce, reverse or prevent the progression of frailty. Notably we suggest that there is a need to challenge stigma around ageing and frailty management; to raise awareness of the malleability of frailty; and to build technology solutions that incorporate social support, or that at least consider the social context, in order to improve health and wellbeing for older adults.

## Conclusion

In conclusion, work must be done to overcome the practical and attitudinal barriers to health care technologies for frailty management before they are perceived as valuable and acceptable to health and social care professionals and will be accepted and used by frail older adults. Given the range of negative stereotypes around frailty and ageing, one challenge is to develop new technologies within a framework that addresses and avoids the stigma surrounding the ‘frail’ label. Rebranding frailty screening and management interventions as resilience building (Bujnowska-Fedak, Gwyther, Szwamel, D'Avanzo, Holland, Shaw and Kurpas: Strategies and beliefs relating to frailty management from the perspectives of stakeholders, forthcoming) or ‘vitality in spite of frailty’ [46], may help to shift public perceptions of frailty and ensure that such interventions are acceptable to the present generation and generations to come. However, this must be accompanied by practical measures to improve access to technology and ICT training for older adults, which would build confidence and provide strategies to address legitimate concerns around the security of personal information.

## Data Availability

The datasets used and/or analysed during the current study may be available from the corresponding author on reasonable request.

## Abbreviations

GP: General Practitioner
ICT: Information and Communication Technologies
PERSSILAA: Personalised ICT Supported Service for Independent Living and Active Ageing
SHARE: Survey of Health, Ageing and Retirement in Europe
